# Current trends and obstacles in off-the-job nursing ethics training in Japanese hospitals: a cross-sectional study

**DOI:** 10.5116/ijme.669f.70b3

**Published:** 2024-08-08

**Authors:** Mari Tsuruwaka

**Affiliations:** 1 Bioethics/Nursing Ethics, Graduate School of Nursing Sciences, St. Luke's International University, Japan

**Keywords:** Continuing education, Nursing ethics training, Off-the-Job Training (OFF-JT), On-the-Job Training (OJT), Japan hospital

## Abstract

**Objectives:**

This study aims to fill the existing gap by examining the current status of off-the-job nursing ethics training in large hospitals in Japan and its integration with on-the-job training to provide targeted insights for enhancing future ethics training.

**Method:**

A cross-sectional study was conducted among the nursing education staff of large Japanese hospitals [N=309] by self-administered questionnaire. The questionnaire was the following main points 1) current trends in nursing ethics training 2) planners’ concerns, and 3) the link between training and clinical practice. Descriptive statistics were used, closed-ended questions were analyzed through simple tabulations while open-ended questions underwent textual analysis.

**Results:**

The hospitals of 76.6% (309) conducted off-the-job nursing ethics training. Their training consists of a combination of lectures and exercises. The focus was to raise nurses’ awareness of ethical problems or improve their analytical ability. The objectives were to be able to participate in discussions from an ethical perspective. The main problems were the lack of connection with on-the-job, and a shortage of training personnel.

**Conclusions:**

The key to providing off-the-job and on-the-job is to create a mechanism for circulation. The implications of the results are the necessity of constructing ethics education in medicine to develop medical professionals who can discuss and act from ethical perspectives. Future research is expected to include the creation of a multidisciplinary ethics training program for the hospital.

## Introduction

Ethical behavior and judgment among nurses are foundational requirements in daily clinical practice, no less in Japan's medical environment, which is characterized by a super-aging society, changes in disease structure, and increasing sophistication of medical technology. Consequently, nursing ethics training has become an important aspect of the basic nursing education curriculum,^1^^,^^2^ making continuing training in nursing ethics for graduate nurses essential. However, integrating such education into on-the-job training (the following is OJT)^3^ has been a challenge. This may be because, in clinical practice, the parties involved tend to have conflicting values and behave emotionally, resulting in complex situations^4^ that require mediation through ethics training.

In Japan, continuing training in nursing ethics has been provided in a variety of settings by medical institutions, medical academic societies, and the Ministry of Health, Labour and Welfare through its “E-FIELD Education for Implementing End-of-Life Discussion program.”^5^ Additionally, the importance of ethics training with a focus on palliative care has been increasingly emphasized in the last few years.^6^ Medical institutions, in particular, have been providing ethics training for a while. Their goal has been to train nurses to approach daily work-related problems ethically.^7^^,^^8^ They also aim to provide nurses with theoretical approaches to nursing ethics and personal development.^9^^,^^10^ Because nurses provide direct health care at the bedside and are usually the first to identify patients' needs, they are often expected to advocate for and support patients, decision-making.^11^^,^^12^

Nursing departments in Japanese hospitals provide nursing skills training, including nursing ethics training. According to a survey of nursing education staff at 407 Japanese hospitals with more than 200 beds across nine prefectures, approximately 86% had conducted nursing ethics training.^13^ Their total training time averaged 2-3 hours, with a more frequent combination of lectures and exercises as participants' years of experience increased. Approximately 56% of hospitals that conducted case studies used problem-solving frameworks. Large-scale hospitals were more likely to offer ethics training, and hospitals with multiple departments were more likely to offer ethics training than those with a single department.

A survey of nurses working in six large-scale hospitals and nine small and medium-size hospitals found that nurses working in large-scale hospitals experienced more ethical problems.^14^ The issues that they most commonly faced can be categorized under “whether patients gave informed consent,” “respect for patients' rights and dignity,” “where does death begin,” “acting against personal and religious values,” and “patient care that may pose a risk to my health.” As in previous research,^13^ educational opportunities were greater in large-scale hospitals, and the levels of recognition of understanding terminology, the need for education, and interest in ethics were also higher in large-scale hospitals. Educational enrichment, including learning about ethical sensitivity, is essential because educational opportunities increase both interest and knowledge about ethical problems, making it easier to understand such problems.^14^ A study on nursing ethics training for new nurses identified concerns such as lack of teaching personnel, problems in securing time for training, and failure to apply learned material in practice.^15^

Further, ethics conferences are held in wards, which are different from off-the-job nursing ethics training. However, repeated ethics conferences are not considered to result in ethical behavior among nurses in clinical settings.^16^ Other challenges include nurses' ability to acquire information, clinical skills such as knowledge of treatment and care, interpersonal skills, and communication skills. These challenges highlight the need for ethics training programs that include teaching practical nursing skills.^16^ Such a program should aim to provide trainees with the knowledge and skills required for each department and to improve communication skills.^16^

Previous research indicates that continuing training in nursing ethics at hospitals is associated with the ethical climate of each hospital.^17^ In addition, nurses' perceptions of ethical problems, ethics training received, and years of nursing experience influenced their perceptions.^18^ Other research reports that continuing training in nursing ethics through on-the-job training prevents moral distress among nurses,^19^ leads to moral behavior,^10^ and improves their ethical decision-making.^20^

This study aims to fill the existing gap in understanding by examining the current status of off-the-job nursing ethics training in large-scale hospitals in Japan and its integration with on-the-job training to provide targeted insights for enhancing future ethics training programs. Therefore, this study aims to provide insights for future continuing training in nursing ethics by clarifying the status of nursing ethics training in large-scale hospitals in Japan and how it is related to on-the-job training.

## Methods

### Study Design and subjects

A cross-sectional study was conducted among the nursing education staff of large Japanese hospitals by a self-administered postal questionnaire. The study targeted 784 hospitals in Japan with over 300 beds. The subjects were nurses in charge of nursing ethics education in the nursing department of a large Japanese hospital, and those who were actually planning nursing ethics training at a hospital.

### Instrument

It judged that a questionnaire survey would be appropriate to clarify the overall trends in nursing ethics training conducted in nursing departments of Japanese hospitals. Since there is no existing questionnaire that meets the purpose of this study, a questionnaire was created for this study.

The questionnaire was created with reference to previous research,^13^^,^^15^ although the size of the hospitals and target groups differed. The questionnaire consisted of closed-ended questions based on the following four items: “basic attributes of participants and hospital size” (years of experience, experience in planning, number of beds), “overview of nursing ethics training in the nursing department” (availability of multiple sessions, conditions for participation, hours of training, pre-training assignments, post-training assignments, training methods, goals, learning outcomes, whether learning outcomes were achieved, points of emphasis, planning issues), “lecture descriptions” (instructor, lecture topics), and “exercise descriptions” (exercise type, exercise strategy, exercise materials, exercise theme, whether a facilitator was present, and whether facilitators were trained). Questions based on the following three items were asked in an open-ended format: “concerns about planning for nursing ethics training,” “concrete ways to translate off-the-job training into on-the-job training,” and “factors necessary for speaking from an ethical perspective.” Based on previous research with new nurses, 15 a questionnaire that focused on an overview of nursing ethics training and the relationship between off-the-job and on-the-job training was developed.

### Data Collection

Questionnaires were sent to education staff in the nursing departments of 784 hospitals with more than 300 beds in government-designated cities (population more than 500,000), core cities (population more than 200,000), and special wards throughout Japan. In Japan, hospitals with more than 300 beds are considered large-scale hospitals. Additionally, for this study, hospitals that have an on-site education department with various plans and procedures in place were considered large. The subjects were those who were actually planning nursing ethics training at a hospital. This survey was conducted between October 2022 and March 2023 among nurse ethics training planners in nursing departments of various hospitals.

### Data Analysis

Descriptive statistics were used. The closed-ended responses were analyzed using simple tabulation while the open-ended responses were analyzed by digitizing and contextualizing the textual data through creating subcategories. In addition, the similarities and common characteristics of the sub-categories were grouped together to form categories at a higher level of abstraction.

The questionnaire was kept anonymous to protect the privacy of the respondents, who were free to decide whether to participate in the survey and could easily express negative opinions. The subjects were provided with an explanation of the purpose, methods, and ethical considerations of the present study, and the voluntariness of the study was ensured. This study was conducted with the approval of the research ethics committee of St. Luke's International University (20A-006).

## Results

### Basic attributes of hospitals and the availability of nursing ethics training

Of the 309 responses received, yielding a 39.4% (784) response rate. Table 1 summarizes the attributes of the participants. The distribution of hospital beds was as follows: 33.0% (309) of the hospitals had 300-399 beds; 54.0% (309), 400-699 beds; 8.1% (309). Ninety percent of hospitals had fewer than 700 beds. Regarding the availability of nursing ethics training (excluding new hires and managers), 76.6% (309) responded with a “yes” and 23.3% (309) with a “no.” The reasons for “no” were “holding ethical conferences on a ward basis”(22.2%(72)), “planning” (20.8%(72)), “can be replaced by ethics training from other departments” (13.9％(72)), and “lack of preparation” (12.5%(72)).

### Overview of nursing ethics training

Table 2 summarizes nursing ethics training. Of the hospitals surveyed, 42.1% (237) had one ethics training course and 56.1% (237) had multiple courses. The conditions for participation were set according to the clinical ladder (for 67.9%: n=237 of the hospital). The most common training duration was 60 to 90 minutes. The requirement for pre-training assignments was approximately 65% or more of hospitals. The most common pre-assignments were “write about a case in which you felt you had an ethical issue” and “write about how you feel about ethics on a daily basis”. The requirement for post-training assignments was approximately 55% or more hospitals. The most common training method was “lectures and exercises”.

The training goals (multiple answers allowed) were as follows, starting with the most common response: “to become aware of ethical problems in clinical practice”, “to learn how to analyze problems that arise in clinical practice from an ethical perspective”, “to examine one's own actions to resolve ethical problems that arise in clinical practice”, “to gain knowledge about ethics in clinical practice”, “to collaborate with a wide variety of professionals to resolve ethical problems in clinical practice”, and “to facilitate, within a team, the resolution of ethical problems that arise in clinical practice”. Concerning whether the learning outcomes were achieved, answered “disagree,” with “agree” and “somewhat agree” together accounting for 72.1% (237) of the total.

The reasons for not achieving the goal were as follows (multiple answers allowed): “training does not translate into clinical practice”, “lecture/training sessions are given in a passive style”, “not enough time is allocated”, “nursing ethics training program in nursing department is not well developed”, “trainees' motivation is low” , “inadequate ethics training on the planning side”, “a lack of qualified personnel”, “a lack of active discussion and debate among participants during exercises”, “does not meet the needs of diverse trainees”, and “the case study selection for exercises was inappropriate”.

The learning outcomes (multiple answers allowed) were “able to participate in discussions about ethical problems and express my thoughts”, “able to participate in discussions about ethical problems and communicate from an ethical perspective”, “able to explain the ethical problems I have experienced to others”, “able to analyze cases from an ethical perspective”, “able to speak from an ethical perspective at a ward conference”, “able to take action to resolve ethical problems that arise in clinical practice”, “able to explain ethical principles”, “able to describe the characteristics of ethical problems in a clinical setting”, “able to collaborate with multiple professions to resolve ethical problems that arise in clinical practice”, “able to explain concepts such as ethics”, and “able to demonstrate leadership in addressing ethical problems in the wards”.

The planner’s emphasis (multiple answers allowed) were “include realistic examples that trainees are likely to encounter”, “create lectures that do not make trainees feel that ethics is a difficult topic to learn”, “enable trainees to experience concrete analysis of ethical issues during training”, “create an environment where trainees can actively participate in discussions and speak up”, “conduct interviews concerning ethical problems that arise in the hospital and apply them to planning”, “include more practical content than ethics and concepts” (10.9%), and “improve areas that trainees rated poorly last year”.

**Table 1:** Overview of respondent and hospitals (n=309)

Question/variable	Responses	Numbers of respondents	％
Are you in charge of education in the nursing department?	Yes	268	86.7
	No	37	12.0
	N.A.	4	1.3
Years pf experiences in clinical settings	1 year to less than 5 years	0	0.0
	5 to less than 10 years	0	0.0
	10 to less than 15 years	4	1.3
	15 to less than 20 years	29	9.4
	20 to less than 25 years	73	23.6
	over 25 years	200	64.7
	N.A.	3	1.0
Experience involved in planning nursing ethics training	less than one year)	37	15.9
	1 year to less than 5 years	112	48.1
	5 to less than 10 years	45	19.3
	10 to less than 15 years	27	11.6
	15 to less than 20 years	9	3.9
	20 to less than 25 years	2	0.9
	over 25 years	0	0.0
	N.A.	5	2.1
Number of beds	300-399 beds	102	33.0
	400-699 beds	167	54.0
	700-999 beds	25	8.1
	more than 1000beds	10	3.2
	N.A.	5	1.6
Is there nursing ethics education as off-the-job training	yes	237	76.6
	no	72	23.3
	N.A.	0	0.0
The reason for "no" (n=72)	holding ethical conferences on a ward basis	16	22.2
	planning	15	20.8
	can be replaced by ethics training from other departments	10	13.9
	lack of preparation	9	12.5
	there is no room for incorporating it into education.	4	5.6

**Table 2:** Overview of nursing ethics training

Question/Variable	Responses	Numbers of respondents	％
Is there only one nursing ethics training? (n=237)	Yes	100	42.1
	No	133	56.1
	N.A.	4	1.6
Participation conditions (n=237)	clinical ladder	161	67.9
	personal preference	31	13.0
	others	25	10.5
	years of experience	13	5.4
	N.A.	7	2.9
Training duration (n=237)	60 minutes	82	34.5
	90 minutes	50	21.0
	120 minutes	36	15.1
	half a day	42	17.7
	a day	7	2.9
	others	19	8.0
	N.A.	1	0.4
pre-training assignments (n=237)	Yes	154	64.9
	No	83	35.0
	N.A	0	0.0
The content for "Yes"（n=154)	write about a case in which you felt you had an ethical issue	81	52.6
	write about how you feel about ethics on a daily basis	18	11.7
	others	49	31.8
	N.A	6	3.9
post-training assignments (n=237)	Yes	131	55.2
	No	104	43.8
	N.A	2	0.8
training method (n=237)	lectures	20	8.4
	lectures and exercises	203	85.6
	exercises	11	4.6
	others	3	1.2
	N.A	0	0.0
training goals (multiple answers) (n=237)	to become aware of ethical problems in clinical practice	139	58.6
	to learn how to analyze problems that arise in clinical practice from an ethical perspective	103	43.4
	to examine one's own actions to resolve ethical problems that arise in clinical practice	70	29.5
	to gain knowledge about ethics in clinical practice	67	28.2
	to collaborate with a wide variety of professionals to resolve ethical problems in clinical practice	27	11.3
	to facilitate, within a team, the resolution of ethical problems that arise in clinical practice	13	5.4
	others	5	2.1
whether the learning outcomes were achieved (n=237)	agree	38	16.3
	somewhat agree	130	55.8
	somewhat disagree	55	23.6
	disagree	1	0.4
	N.A.	13	5.6
The reasons for not achieving the goal (multiple answers)(n=56)	training does not translate into clinical practice	35	62.5
	lecture/training sessions are given in a passive style	33	58.9
	not enough time is allocated	23	41.1
	nursing ethics training program in nursing department is not well developed	22	39.3
	trainees' motivation is low	21	37.5
	inadequate ethics training on the planning side	13	23.2
	a lack of qualified personnel	10	17.9
	a lack of active discussion and debate among participants during exercises	6	10.7
	does not meet the needs of diverse trainees	5	8.9
	the case study selection for exercises was inappropriate	1	1.8
	others	4	7.1
The learning outcomes(multiple answers)(n=237)	able to participate in discussions about ethical problems and express my thoughts	109	45.9
	able to participate in discussions about ethical problems and communicate from an ethical perspective	82	34.5
	able to explain the ethical problems I have experienced to others	79	33.3
	able to analyze cases from an ethical perspective	78	32.9
	able to take action to resolve ethical problems that arise in clinical practice	53	22.3
	able to speak from an ethical perspective at a ward conference	56	23.6
	able to explain ethical principles	47	19.8
	able to describe the characteristics of ethical problems in a clinical setting	32	13.5
	able to collaborate with multiple professions to resolve ethical problems that arise in clinical practice	27	11.1
	able to explain concepts such as ethics	24	10.1
	able to demonstrate leadership in addressing ethical problems in the wards	16	6.7
	others	8	3.3
Lecturers (n=225)	certified nurse specialists	82	36.4
	education staff in a nursing department	72	32.0
	personnel in a hospital who are familiar with nursing ethics	29	12.8
	head nurses of a ward	27	12.0
	deputy directors of a nursing department	25	11.1
	external ethics experts	13	5.7
	internal ethics experts	3	1.3
	others	64	28.4
The subjects of the lectures(n=225)(multiple answers)	what is nursing ethics?	174	77.3
	ethical problems and dilemmas faced in clinical practice	169	75.1
	what is ethics?	161	71.5
	ethical principles	160	71.1
	JNA Code of Ethics for Nurses and other codes of ethics	144	64.0
	ethical responsibility and ethical behavior of nurses	133	59.1
	approach to considering ethical problems	131	58.2
	methodology for examining ethical problems	114	50.6
	rights of patients	110	48.8
	significance of discussing ethical problems	108	48.0
	points to consider when examining ethical problems	101	44.8
	autonomy of patients	81	36.0
	informed consent	69	30.6
	confidentiality	63	28.0
	protection of patients' personal information	60	26.6
	others	13	5.7
Form of exercise （n=215）	small group work	201	93.4
	role-playing	4	1.8
	others	9	4.1
	N.A	1	0.4
he degree to which they devised the exercises (n=215)	devising	135	62.7
	somewhat devising	57	26.5
	not devising much	7	3.7
	not devising	1	0.9
	N.A.	15	6.0
The methods they used for devising (multiple answers) (n=192)	tell trainees that it is important to be able to discuss ethical problems and feelings of discomfort with other trainees in lectures	144	75.0
	tell trainees to express their own thoughts and ideas during exercises	140	72.9
	consider group composition so that trainees can actively speak up	135	70.3
	tell trainees that speaking from an ethical perspective will lead to the welfare of patients in practice	61	31.8
	others	29	15.1
materials used in the exercises (n= 215)	common simulated cases	99	45.5
	cases experienced by trainees	76	35.3
	cases in cartoons	5	2.3
	video materials	3	1.8
	others	12	5.1
	N.A.	21	9.7
The selection of case studies(n= 215)(multiple answers)	suggested by the planner	104	48.3
	selected based on the cases in the pre-assignment	35	16.2
	selected from trainees' experiences	35	16.2
	selected themes under discussion in the hospital	10	4.6
	selected from themes requested by trainees	9	4.1
	selected themes popular in society	2	0.9
	others	18	8.3
	N.A	2	0.4
The details of the cases(n=215)(multiple answers)	physical restraint	128	59.5
	treatment choice and decision making for patients with capacity	120	55.8
	surrogate decision-making for patients with diminished decision-making capacity	121	56.2
	matters related to the intentions of patients and families and their differences	134	62.3
	matters related to respect for patients’ rights	95	44.1
	matters related to end-of-life care	87	40.4
	matters related to conflicts of values with physicians and other professions	81	37.6
	matters related to the personality and dignity of patients	76	35.3
	informed consent	50	23.2
	matters related to confidentiality and personal information	31	14.4
	others	12	5.5
Facilitators to support exercises (n=215)	Yes	158	73.4
	No	56	26.0
	N.A.	1	0.4
Continuing training for facilitators (n=158)	Yes	28	17.7
	No	130	82.2
	N.A.	0	0.0
Self-improvement as a planner (n=237)	Have outside learning opportunities to improve knowledge and skills related to nursing ethics	114	48.1
	Gather new information about nursing ethics through books and the internet	87	36.7
	Participate in internal training to improve knowledge and skills regarding nursing ethics	12	5.0
	nothing	9	3.7
	others	7	2.9
	N.A	8	3.3
The planner’s emphasis(multiple answers)(n=237)	create lectures that do not make trainees feel that ethics is a difficult topic to learn	103	43.4
	include realistic examples that trainees are likely to encounter	111	46.8
	enable trainees to experience concrete analysis of ethical issues during training	87	36.7
	create an environment where trainees can actively participate in discussions and speak up	78	32.9
	conduct interviews concerning ethical problems that arise in the hospital and apply them to planning	30	12.6
	include more practical content than ethics and concepts	26	10.9
	improve areas that trainees rated poorly last year	10	4.2
	others	5	2.1
Challenges by planners (multiple answers)(n=237)	training does not translate into practice	96	40.5
	there is a lack of personnel to provide training	76	32.0
	there is no continuing training program	62	26.1
	lectures / training sessions are delivered in a passive style	61	25.7
	not enough time is allocated	59	24.8
	ethics training on the planning side is inadequate	51	21.5
	there level of ethics awareness within the organization is not high	48	20.2
	participation from other professions is lacking	40	16.8
	trainees' motivation is low	38	16.0
	trainees cannot express or discuss ethical problems during exercises	10	4.2
	does not meet the needs of diverse trainees	8	3.3
	the selection of case studies for exercises was inappropriate	4	1.6
	the training method was inappropriate	2	0.8
	others	20	8.4

Challenges by planners raised by planners (multiple answers allowed) were “training does not translate into practice”, “there is a lack of personnel to provide training”, “there is no continuing training program”, “lectures / training sessions are delivered in a passive style, “not enough time is allocated”, “ethics training on the planning side is inadequate”, “there level of ethics awareness within the organization is not high”, “participation from other professions is lacking”, “trainees' motivation is low”, “trainees cannot express or discuss ethical problems during exercises”, “does not meet the needs of diverse trainees”, “the selection of case studies for exercises was inappropriate”, and “the training method was inappropriate”.

### Lectures and Exercises

Most of the lecturer were certified nurse specialists in a hospital, followed by education staff in a nursing department.

The subjects of the lectures (multiple answers allowed) were “what is nursing ethics?”, “ethical problems and dilemmas faced in clinical practice”, “what is ethics?”, “ethical principles”, “JNA Code of Ethics for Nurses and other codes of ethics”, “ethical responsibility and ethical behavior of nurses”, “approach to considering ethical problems”, “methodology for examining ethical problems”, “rights of patients”, “significance of discussing ethical problems”, “points to consider when examining ethical problems”, “autonomy of patients”, “informed consent”, “confidentiality”, and “protection of patients' personal information”.

Of the 215 hospitals that included exercises in their training, most of them employed “small group work”. Concerning the degree to which they devised the exercises, “devising” and “somewhat devising” accounting for about 90% of the respondents. The methods (multiple answers allowed) they used for devising were “tell trainees that it is important to be able to discuss ethical problems and feelings of discomfort with other trainees in lectures”, “tell trainees to express their own thoughts and ideas during exercises”, “consider group composition so that trainees can actively speak up”, and “tell trainees that speaking from an ethical perspective will lead to the welfare of patients in practice”.

The most common type of materials used in the exercises was “common simulated cases”, followed by “cases experienced by trainees”. The most common type of selection of case studies was “suggested by the planner”.

The details of the cases (multiple answers allowed) were “matters related to the intentions of patients and families and their differences”, “physical restraint”, “surrogate decision-making for patients with diminished decision-making capacity”, “treatment choice and decision making for patients with capacity”, “matters related to respect for patients’ rights”, “matters related to end-of-life care”, “matters related to conflicts of values with physicians and other professions”, “matters related to the personality and dignity of patients”, “informed consent”, and “matters related to confidentiality and personal information”.

Concerning whether a facilitator was present during the exercise, 73.4% (215) responded “yes” and 26.0% (215), “no.” Regarding whether facilitators were trained in the hospital, 17.7% (158) responded “yes” and 82.2% (158), “no.”

### Concerns about planning nursing ethics training

Concerning whether they had concerns about training planning, 39.2% (237) of the respondents answered “yes;” 33.7% (237), “somewhat;” 19.8% (237), “hardly;” and 1.2% (237), “no.” Those who responded with “yes” and “somewhat” together accounted for 72.9% of the respondents. Details of concerns were noted in an open-ended manner. They were divided into four themes: the planning side, issues related to planning, the trainee's side, and translation into practice (Table 3).

Concerns about competence included “planners’ lack of competence in ethics,” “differences in trainees’ ethics competence,” and “lack of ethics experts in the organization.” Concerns about the system included “unable to secure staff to focus on training,” “one-person system with no one to consult, ” “burdened by the time required for preparation, ” “high turnover of training staff on an annual basis, ” “no ongoing training to improve skills, ” and “unable to secure facilitators. ” For concerns related to planning, nine categories and 26 subcategories were extracted. The nine categories are lecture topics, sharing opinions and progress, trainees and level settings, goal, learning outcomes, selection of case studies, selection of instructors and ensuring that they are well qualified, selection of case study analysis methods, and training hours.

Concerns about lectures were “how to teach things that don't have answers?” “Topics are difficult,” and “stuck in a rut,” which were influenced by the nature of ethics. Concerns about sharing opinions and progress were “creating an atmosphere that acknowledges different values,” “providing an opportunity to become aware of everyday ethics,” “presentations and sharing of opinions tend to be redundant,” “discussions do not progress well” and “facilitation is inadequate.” Concerns about the trainees and level settings were “multi professions are not participating,” “whether the training is appropriate for each clinical ladder,” and “not being able to meet the needs of each trainee."

Concerns about goals were “where to set the theme,” “there is a mismatch between goals and learning content,” and “the level of difficulty is set too low.” Concerns about learning outcomes were “where to focus” and “cannot achieve learning outcomes.”

Concerns about the selection of case studies were “whether they are appropriate as examples,” “similar examples are used,” “privacy should be taken into consideration,” and “whether on-site problems can be presented.” Concerns about selection of instructors and ensuring they are qualified were “whether the qualifications of instructors are appropriate” and “ensuring that instructors are well versed in ethics.” Concerns about the selection of case study analysis methods were “whether effective methods are selected” and “difficulty in deepening understanding of analysis methods.” Concerns about the training hours were “too short and the content is not conveyed” and “lack of time for exercises.”

For concerns about trainees, five categories and 12 subcategories were extracted. The five categories were differences in trainee readiness, large knowledge gaps, value differences in the planning side, passive attitude, and a tendency to find answers too quickly. Concerns about differences in trainee readiness were “belong to different departments,” “become less motivated each year,” “few participants,” and “perceive that ethics is difficult.” Concerns about large knowledge gaps were “difficulty capturing knowledge in advance” and “inconsistent levels of knowledge.” Concerns about value differences in the planning side were related to differences in sensibilities and generational values, such as “how to appeal to the sensibilities of Generation Z?” and “not feeling a dilemma.” Concerns about passive attitudes were “not speaking up or expressing an opinion” and “just listening to the lecture.” Concerns about a tendency to seek answers were “sticking to the right answer” and “trying to find answers too quickly.”

Regarding translation into clinical practice, three categories and six subcategories were extracted. The three categories are “the training is not translated into clinical practice,” “there is no system to apply the learning in training to clinical practice,” and “the use of training in practice cannot be evaluated.” Concerns that the training is not translated into clinical practice were the “content of the training is not clinically applicable” and “concepts and clinical practice cannot be integrated.” Concerns that there was no system for applying the material learned during training to clinical practice were “ethics conferences in wards are not taking root” and “not used to discussing ethics.” Concerns related to the use of the training in practice not being evaluated were “unable to identify clinical uses in detail” and “no method to assess specific uses.”

### Concrete ways to link off-the-job training to clinical practice

Respondents were asked to write in an open-ended format about the details of how they had linked off-the-job nursing ethics training to clinical practice. Table 4 shows the results. Eight categories and 25 subcategories were extracted. The eight categories were “assigning tasks in the clinical setting after training,” “unification of analysis tools in clinical practice and training,” “participation of planners in clinical practice,” “regularizing and promoting ethics conferences in each department,” “a system for applying the learning from the training within the department,” “organization-wide efforts to strengthen ethics,” “planning for practice-based education,” and “series-based, step-by-step planning.”

**Table 3:** Concerns about planning nursing ethics training

Item to consider		Concerns
planning side	competence	planners’ lack of competence in ethics
		differences in trainees’ ethics competence
		lack of ethics experts in the organization
	system	unable to secure staff to focus on training
		one-person system with no one to consult
		burdened by the time required for preparation
		high turnover of training staff on an annual basis
		no ongoing training to improve skills
		unable to secure facilitators
planning	lecture topics	how to teach things that don't have answers?
		topics are difficult
		stuck in a rut
	sharing opinions and progress	creating an atmosphere that acknowledges different values
		providing an opportunity to become aware of everyday ethics
		presentations and sharing of opinions tend to be redundant
		discussions do not progress well
		facilitation is inadequate
	trainees and level settings	multi professions are not participating
		whether the training is appropriate for each clinical ladder
		not being able to meet the needs of each trainee
	goal	where to set the theme
		there is a mismatch between goals and learning content
		the level of difficulty is set too low
	learning outcomes	where to focus
		cannot achieve learning outcomes
	selection of case studies	whether they are appropriate as examples
		similar examples are used
		privacy should be taken into consideration
		whether on-site problems can be presented
	selection of instructors and ensuring that they are well qualified	whether the qualifications of instructors are appropriate
		ensuring that instructors are well versed in ethics
	selection of case study analysis methods	whether effective methods are selected
		difficulty in deepening understanding of analysis methods
	training hours	too short and the content is not conveyed
		lack of time for exercises
trainee's side	differences in trainee readiness	belong to different departments
		become less motivated each year
		few participants
		perceive that ethics is difficult
	large knowledge gaps	difficulty capturing knowledge in advance
		inconsistent levels of knowledge
	value differences in the planning side	how to appeal to the sensibilities of Generation Z?
		not feeling a dilemma
	passive attitude	not speaking up or expressing an opinion
		just listening to the lecture
	a tendency to find answers too quickly	sticking to the right answer
		trying to find answers too quickly
translation into practice	the training is not translated into clinical practice	content of the training is not clinically applicable
		concepts and clinical practice cannot be integrated
	there is no system to apply the learning in training to clinical practice	ethics conferences in wards are not taking root
		not used to discussing ethics
	the use of training in practice cannot be evaluated	unable to identify clinical uses in detail
		no method to assess specific uses

**Table 4:** Concrete ways to link off-the-job training to clinical practice

Item to consider	Link to clinical practice setting
assigning tasks in the clinical setting after training	trainees planning ethics conferences in wards
	trainees submitting reports after ethics conferences in wards
unification of analysis tools in clinical practice and training	using analytic tools that are suitable for clinical practice
	using reflection notes to facilitate conferences
participation of planners in clinical practice	planner attendance at ward conferences
	support and comment on conference preparation
regularizing and promoting ethics conferences in each department	include ethical issues in case studies from wards for ethics conferences regularly
	discuss experiences from training at conferences in our department
	hold regular ethics conferences in each department
	make ethics conferences regular in each department
a system for applying the learning from the training within the department	creating opportunities to apply the outcomes of the training in collaboration with nurse managers
	assigning clinical roles to trainees based on the training
	working with ethics education staff in each department
organization-wide efforts to strengthen ethics	hold study sessions for physicians and executives
	show how to conduct conferences and demonstrate the significance of conference
	share educational information with other committees and nurse managers
	develop personnel to be familiar with ethics in the department
	open and share training information
planning for practice-based education	try an ethics conference during training
	use commonly encountered cases in training
	use an example from our department in training
	provide training on the management and use of ethics conferences
series-based, step-by-step planning	create a plan to step up
	make it a series
	create a plan that all employees can participate in throughout the year

For assigning tasks in the clinical setting after training, the translation of the learned material in training into clinical practice was found through “trainees planning ethics conferences in wards” and “trainees submitting reports after ethics conferences in wards.” For unification of analysis tools in clinical practice and training, the methods for applying the analysis methods learned in training to clinical practice were “using analytic tools that are suitable for clinical practice” and “using reflection notes to facilitate conferences.”

Ways for planners to participate in and connect with clinical practice were “planner attendance at ward conferences” and “support and comment on conference preparation.”

Regularizing and promoting ethics conferences in each department comprised “include ethical issues in case studies from wards for ethics conferences regularly,” “discuss experiences from training at conferences in our department,” “hold regular ethics conferences in each department,” and “make ethics conferences regular in each department.”

A system for applying the material learned during training within the department included “creating opportunities to apply the outcomes of the training in collaboration with nurse managers,” “assigning clinical roles to trainees based on the training,” and “working with ethics education staff in each department.” Organization-wide efforts to strengthen ethics were “hold study sessions for physicians and executives,” “show how to conduct conferences and demonstrate the significance of conference,” “share educational information with other committees and nurse managers,” “develop personnel to be familiar with ethics in the department,” and “open and share training information.”

Planning for practice-based training included “try an ethics conference during training,” “use commonly encountered cases in training,” “use an example from our department in training,” and “provide training on the management and use of ethics conferences.” Series-based, step-by-step planning included “create a plan to step up,” “make it a series,” and “create a plan that all employees can participate in throughout the year.”

### Factors that enable ethical communication

Respondents were asked about the aspects that they thought could facilitate communication from an ethical perspective (Table 5). Seven categories and 22 subcategories were extracted. The seven categories were patient-centeredness, interpersonal communication and conference management skills, continuing training for broad knowledge acquisition, re-education of nurse managers and site leaders, a system for ethical awareness and collaboration in clinical practice, a place to talk about nursing, and a workplace climate that recognizes diverse values.

**Table 5:** Factors that enable ethical communication

Item to consider	Factors enabling ethical communication
patient-centeredness	speak from a patient-centered perspective
	recognize my role as an advocator
	understand patient needs
interpersonal communication and conference management skills	undergo training in conference management
	undergo facilitation training to bring out individual value
	develop the ability to express oneself assertively
	be able to relate to others in a compassionate way
continuing training for broad knowledge acquisition	attending external training
	exchanging views with staff from other institutions
	improving basic skills as a member of society
	taking an interest in social affairs
re-education of nurse managers and site leaders	strengthen basic ethics education for site leaders
	develop facilitation skills for site leaders
	create role models
a system for ethical awareness and collaboration in clinical practice	having a place to think about ethical problems on a regular basis
	thinking about ethical issues using familiar cases
	having cross-functional relationships across departments
	undergoing teamwork training
a place to talk about nursing	having the opportunity to reflect on and talk about one's own nursing experience
	having the opportunity to communicate with each other about daily concerns
	having leaders and senior staff talk about nursing
a workplace climate that recognizes diverse values	no one is criticized for expressing an opinion
	the workplace is psychologically safe
	there are opportunities to talk with other professions as well as nursing staff
	conversations are encouraged throughout the organization

Patient-centeredness was thought to help nurses speak in an ethical manner. It was considered necessary to “speak from a patient-centered perspective,” “recognize my role as an advocator,” and “understand patient needs.”

Having interpersonal communication and conference management skills was also considered important for ethical communication. For example, it was considered necessary to “undergo training in conference management,” “undergo facilitation training to bring out individual value,” “develop the ability to express oneself assertively,” and “be able to relate to others in a compassionate way.”

Continuing training that was considered necessary for the acquisition of a wide range of knowledge was “attending external training,” “exchanging views with staff from other institutions,” “improving basic skills as a member of society” and “taking an interest in social affairs.”

For the re-education of nurse managers and site leaders, it was considered necessary to “strengthen basic ethics education for site leaders,” “develop facilitation skills for site leaders,” and “create role models.”

A system for ethical awareness and collaboration in clinical practice that was considered necessary was “having a place to think about ethical problems on a regular basis,” “thinking about ethical issues using familiar cases,” “having cross-functional relationships across departments,” and “undergoing teamwork training.”

Opportunities to talk about nursing that were considered necessary were “having the opportunity to reflect on and talk about one's own nursing experience,” “having the opportunity to communicate with each other about daily concerns,” and “having leaders and senior staff talk about nursing.”

Workplace climate that recognizes diverse values that were considered necessary were “no one is criticized for expressing an opinion,” “the workplace is psychologically safe,” “there are opportunities to talk with other professions as well as nursing staff,” and “conversations are encouraged throughout the organization.

## Discussion

The aim of this study was to clarify the current status of off-the-job training in nursing ethics education at large Japanese hospitals. Eighty percent of large-scale hospitals in Japan conduct nursing ethics training as off-the-job training; many of them actively plan multiple training courses. Compared to three previous studies,^13^^-^^15^ the training hours in this study tended to be shorter. More than 70% of the respondents in this study used facilitators in their exercises, but of these, more than 80% did not have in-hospital training.

The barrier to nursing ethics education training, which was the aim of this study, was revealed to be the problem of human resources on the training side. Prior to examining the issues of planning, the study revealed that the problem, which has been pointed out in previous research,^15^ is planners’ lack of knowledge and skills regarding ethics. In other words, training programs are planned even though the planners do not feel that they have sufficient planning skills. In addition, the planners in the study had to plan alone, were replaced every year, and did not have access to a consultation system. Further, there was little training for exercise facilitators. Planning ethics training, unlike planning nursing skills training, requires different skills outside of nursing. It requires knowledge of ethics and the ability to identify clinical issues and respond to needs. The first step in planning is to establish a system for consulting experts and professionals of ethics, including outside specialists, to plan and manage training.

The results of this study suggest the importance of collaboration and integration of off-the-job and on-the-job training, which is necessary to improve the quality of ethics education training within hospitals. Most respondents felt that it was important to link off-the-job nursing ethics training with on-the-job training. To facilitate this connection, many hospitals combined lectures and exercises and took steps such as using examples commonly encountered in clinical practice. However, respondents recognized that they were not sufficiently connected. In Japan, ethics training has traditionally been conducted as a project of a hospital’s nursing department. However, to reproduce the clinical context in training, they need to plan together with other professions and create a training environment where their values conflict with those of other professions, as in the clinical setting.

It is important to keep in mind that off-the-job and on-the-job training are always a two-way cycle, rather than off-the-job training preceding on-the-job training. As indicated in this study, the key was to create a mechanism for circulation, such as having planners attend clinical ethics conferences, developing nurse liaisons to address ethics issues and sharing staff with the nursing education department, as well as investigating how ethics conferences should be conducted in a clinical setting and what analytical tools should be used by both sides. These mechanisms were suggested to be implemented as part of creating an ethical climate within the organization rather than within the nursing department. Previous research has also shown that there is a relationship between ethics training and the ethical climate of organizations.^17^

This study focused on the verbalization of ethical problems as a goal of the training. In nursing ethics training, each person deepens his or her ethical thinking, speaks from an ethical perspective, and discusses with others. Through these actions, they find approaches to advocate for the rights of patients. The results of this study show that to achieve this, it was crucial to improve nurses’ ability to talk about nursing and discuss it with people from different professions regularly. Off-the-job nursing ethics training is not simply about acquiring knowledge and deepening ethical thinking. The study results suggest that it is also important for trainees to be able to speak and act from an ethical perspective to achieve the best outcomes for patients.

This study was able to clarify the overall picture and issues of nursing ethics training in nursing departments of Japanese hospitals. Although each hospital offered multiple nursing ethics training, such as for new nurses and nursing managers, this study analyzed only one nursing ethics training course, which was attended by many nursing staff. Further research is needed in the future to examine analysis of the structure and relationships of multiple ethics training programs at large Japanese hospitals.

## Conclusions

This study aimed to fill the existing gap in understanding by examining the current status of off-the-job nursing ethics training in large-scale hospitals in Japan and its integration with on-the-job training to provide targeted insights for enhancing future ethics training programs. Eighty percent of large-scale hospitals in Japan conduct nursing ethics training as off-the-job training and provide multiple training courses. To ensure that both off-the-job and on-the-job training were well connected, many hospitals combined lectures and exercises and used examples commonly encountered in clinical practice, among other strategies. However, participants recognized that they were not sufficiently connected. The key to off-the-job and on-the-job training is to create a mechanism for circulation. Suggestions include making an organization-wide effort to strengthen ethics and planning ethics training with other professions. Regarding planners’ lack of competence, it is crucial to establish a system for consulting experts and professionals of ethics, including external specialists. The implications of the results are the necessity of constructing practical ethics education in medicine and nursing to develop medical professionals who can discuss and act from an ethical perspective in clinical settings. Future research is expected to include the creation of a multidisciplinary ethics training program for the hospital, rather than just a nursing department.

### Acknowledgements

I would like to thank the participants in this study. This Study was supported by the Kitano Foundation of Life-long Integrated Education in 2019.

### Conflict of Interest

The author declares that there is no conflict of interest.
